# Understanding effects in reviews of implementation interventions using the Theoretical Domains Framework

**DOI:** 10.1186/s13012-015-0280-7

**Published:** 2015-06-17

**Authors:** Elizabeth A. Little, Justin Presseau, Martin P. Eccles

**Affiliations:** Institute of Health and Society, Newcastle University, Baddiley-Clark Building, Richardson Road, Newcastle Upon Tyne, NE2 4AX UK

**Keywords:** TDF, Behaviour change interventions, Systematic review, Osteoporosis, Fragility fracture, BMD scanning, Bisphosphonates, Secondary analysis

## Abstract

**Background:**

Behavioural theory can be used to better understand the effects of behaviour change interventions targeting healthcare professional behaviour to improve quality of care. However, the explicit use of theory is rarely reported despite interventions inevitably involving at least an implicit idea of what factors to target to implement change.

There is a quality of care gap in the post-fracture investigation (bone mineral density (BMD) scanning) and management (bisphosphonate prescription) of patients at risk of osteoporosis. We aimed to use the Theoretical Domains Framework (TDF) within a systematic review of interventions to improve quality of care in post-fracture investigation. Our objectives were to explore which theoretical factors the interventions in the review may have been targeting and how this might be related to the size of the effect on rates of BMD scanning and osteoporosis treatment with bisphosphonate medication.

**Methods:**

A behavioural scientist and a clinician independently coded TDF domains in intervention and control groups. Quantitative analyses explored the relationship between intervention effect size and total number of domains targeted, and as number of different domains targeted.

**Results:**

Nine randomised controlled trials (RCTs) (10 interventions) were analysed. The five theoretical domains most frequently coded as being targeted by the interventions in the review included “memory, attention and decision processes”, “knowledge”, “environmental context and resources”, “social influences” and “beliefs about consequences”. Each intervention targeted a combination of at least four of these five domains. Analyses identified an inverse relationship between both number of times and number of different domains coded and the effect size for BMD scanning but not for bisphosphonate prescription, suggesting that the more domains the intervention targeted, the lower the observed effect size.

**Conclusions:**

When explicit use of theory to inform interventions is absent, it is possible to retrospectively identify the likely targeted factors using theoretical frameworks such as the TDF. In osteoporosis management, this suggested that several likely determinants of healthcare professional behaviour appear not yet to have been considered in implementation interventions. This approach may serve as a useful basis for using theory-based frameworks such as the TDF to retrospectively identify targeted factors within systematic reviews of implementation interventions in other implementation contexts.

**Electronic supplementary material:**

The online version of this article (doi:10.1186/s13012-015-0280-7) contains supplementary material, which is available to authorized users.

## Background

Changing the behaviour of healthcare professionals is key to achieving the goals of evidence-based medicine, ensuring that research findings are translated into clinical practice and the best possible outcomes for patients are realised. This is not an easy task to carry out systematically, routinely and uniformly, but it has been shown to be more effective if interventions are based on evidence-based principles drawn from theories of behaviour and behaviour change [1, 2].

Theory can be used prospectively to identify and describe the processes involved in existing patterns of care and the barriers and facilitators to change that could be targeted by an intervention [3, 4]. Theory can then be used to guide the development and content of the intervention, giving an understanding of how and why the intervention is designed to work and a framework for testing the mechanism(s) underlying behaviour change [5–7]. The explicit use of theory in this way not only provides a generalizable framework facilitating the replication of interventions in other settings [8], but there is also evidence that behaviour change interventions informed by theory are more effective than those that are not [1, 2, 9]. Despite increasing recognition that the design of behaviour change interventions should be based on relevant theories [8, 10, 11], systematic reviews of behaviour change interventions applied to change the behaviour of healthcare professionals show that theory is rarely used to explicitly underpin intervention methods [12]. Thus, even if positive effects are reported, we are left with little understanding of the behaviour change processes responsible, no basis for choosing between different types of intervention, and little to inform the design of future interventions.

Although interventions may not always be based on a theory explicitly, it is inevitable that any intervention designer will have at least an implicit idea of what factors to target to promote change. Such implicit theories may be helpful for the specific setting in which they are used, but without making them explicit, they cannot be tested and replicated. Given the likely preponderance of implicit theories, it may be useful to retrospectively identify which factors implementation interventions report targeting and the extent to which such factors map onto pre-existing theoretical factors. Such a process would be inferential but would nevertheless provide a better capacity to identify what factors seem to be targeted and thus contribute to a cumulative evidence base for designing future interventions.

Retrospective coding of targeted factors requires a sufficiently broad framework of theoretical factors to capture the potential range of possible targeted factors. The Theoretical Domains Framework (TDF) [13] was developed and validated [14] to summarise the array of psychological theory underpinning behaviour change into distinct factors.

The TDF provides a useful basis for both assessing implementation problems and for identifying potential modifiable factors to target when designing interventions [15]. It is increasingly used as a tool for conducting interviews and designing questionnaires [16–20], but it may also be used as a basis for understanding what factors may have been targeted for change in an intervention. This has the potential to enhance understanding of the behaviour change processes inherent in the transfer of evidence-based guidelines into practice, but to our knowledge, the TDF has yet to be applied in this way. Using the clinical setting of post-fracture investigation and management of patients at risk of osteoporosis, we aimed to conduct a secondary analysis of a systematic review using the TDF to identify the factors targeted by the interventions.

### Post-fracture management of osteoporosis risk

The post-fracture management of patients at risk of osteoporosis is an area of patient care in which there is evidence of a large quality of care gap [21–30] despite widely available evidence-based guidelines [31–35]. Two key post-fracture management behaviours for which a quality gap has been widely documented include primary and secondary healthcare professionals scanning bone mineral density and prescribing anti-resorptive therapy.

We conducted a systematic review of interventions (reported elsewhere) [36] aiming to improve the investigation and management of osteoporosis in patients following fragility fracture. For both of the main outcomes of the review (bone mineral density (BMD) scanning and osteoporosis treatment with anti-resorptive therapy), all nine identified studies [37–45] reported a positive effect of the intervention, with an overall 36 % absolute increase in BMD scanning rates and a 20 % absolute increase in treatment rates.

Whilst the review provided a compelling case that implementation interventions can be effective in improving scanning and treatment rates, replicability of these interventions is undermined by a lack of capacity to understand how the intervention had its effect, i.e. what factors did the interventions target to promote change.

The aim of this paper was to explore, using the TDF, the factors that the interventions may have been targeting, how this might be related to the main outcomes of the systematic review (rates of BMD scanning and osteoporosis treatment with bisphosphonate medication) and highlighting opportunities for targeting factors that seem to be less frequently considered but may be worth exploring in future interventions.

## Methods

### Description of interventions

As part of the systematic review, a verbatim description of the interventions as delivered and the care received by the control group was extracted and reported. The authors of the studies included in the systematic review were contacted by email and informed of our intention to carry out a secondary analysis of their interventions. It was requested in the email that firstly they verify whether or not they agreed with the details of their intervention which were identified and described in the email and secondly send us any additional materials that were given to those delivering the intervention or any protocols or linked studies that described the intervention, how it was carried out and what it was targeting, in more detail. Those nominated as the corresponding author were contacted initially, but if the email address was no longer functioning, alternative authors were tried. A first reminder was sent 2 weeks after the initial email, a second reminder 2 weeks after that, and the authors were then given a further 2 weeks to respond.

### Coding of the TDF domains targeted by the interventions

We used the 14 domains from TDFv2 [14] as a basis for coding TDF domains. The TDF domains that appeared to be targeted by the interventions and within the control groups were identified and coded independently by two reviewers (EAL, a clinician and JP, a behavioural scientist), using a data extraction form designed for the purpose (Additional file 1). We used domains as well as constructs within domains to inform coding decisions within domains, using construct definitions as described by Cane et al. [14]. The data extraction form was tested on one included study. The coding of each domain was supported by evidence from the text. However, this required inferences to be made about which domains the authors were intending to target, as this was never explicitly stated in the text. The descriptions of the interventions were studied and each aspect that was judged to be targeting a domain with respect to the behaviours of BMD scanning and osteoporosis treatment with anti-resorptive medication was coded. The domains were also coded according to the individual targeted, i.e. patient or healthcare professional. Inter-rater reliability was calculated prior to resolving discrepancies (Cohen’s kappa: *Κ* = 0.507 (moderate agreement [46]). Following discussion with a third party (MPE), 100 % agreement was achieved.

### Quantitative analysis

The relationship between the total number of domains coded and effect size of the intervention was explored using Pearson correlations (two-tailed) for both BMD scanning and osteoporosis treatment with anti-resorptive therapy. “Total number of domains” is a sum of every time any domain was coded and does not take into account the recipient of the intervention. For example, if “knowledge” was coded twice for the primary care physician (PCP) and once for the patient, and “beliefs about consequences” was coded three times for the PCP, this would add up to a total of six times. The total number of times the domains were coded in the control groups of the studies was subtracted from the total number of times the domains were coded in the intervention groups. This was a subtraction of total number of domains and did not take into consideration which domains were coded in each group. To investigate whether subtracting domains that appear in the control group impacted on the findings, we conducted a sensitivity analysis whereby all domains coded in the intervention group were counted irrespective of whether also coded in the control group.

The relationship between the number of *different* domains coded and the effect size of the intervention was also explored using Pearson correlations for both BMD scanning and osteoporosis treatment with anti-resorptive therapy. The recipient of the intervention was not taken into account. For example, if “knowledge” was coded five times, “skills” once and “social influence” twice, this would mean that three different domains had been coded. The maximum possible number of different domains coded was 14 (the number of TDF domains). The number of different domains coded in the control group was subtracted from the number of different domains coded in the intervention group. A sensitivity analysis was performed in which the subtraction of control groups was not done, to examine the effect of the subtraction on the result.

## Results

### Descriptions of the interventions and control care

With regard to the development of the intervention, only two studies reported consulting with representatives of the intended professional recipients [40, 43]. Six studies reported the evidence base for the intervention [37, 38, 40–43]. Patient involvement was not reported by any of the studies. In four studies, the authors reported specific barriers to change that the intervention was tailored to address [37, 40, 42, 44]. Only one study reported that they had carried out substantial exploratory work to identify them [40] (literature reviews and qualitative in-depth interviews with healthcare professionals). In two of the four studies, barriers were not identified and discussed until the end of the paper [37, 44]. Only one study designed the intervention to address all of the identified barriers [40]; the others offered no explanation as to why they had chosen particular barriers to target over others, with one study selecting a single barrier from a long list of barriers [44].

The coding for the domains targeted in the intervention and control groups for each of the studies is shown in Table 1. A more detailed description of the coding as well as the care given to intervention and control groups by study is presented in Additional file 2.

**Table 1:** Coding of domains targeted in the intervention and control groups

Study	Intervention group: domains targeted	Control group: domains targeted
Gardner 2005	Patient	None identified
	• Knowledge (knowledge from 15-min educational visit)	
	• Beliefs about consequences (T: effectiveness of therapies)	
	• Environmental context and resources (material resource of questions)	
	• Memory, attention and decision processes (attention from telephone call at 6 weeks)	
	• Beliefs about consequences (call may have targeted beliefs about consequences of seeking follow-up with PCP)	
	PCP	
	• Goals (questions are goals and action plans)	
	• Memory, attention and decision processes (questions focus attention)	
	• Memory, attention and decision processes (attention from patient attending to discuss management of osteoporosis)	
Feldstein 2006	Intervention 1	None identified
	PCP	
	• Knowledge (knowledge from guidelines)	
	• Memory, attention and decision processes (attention from EMR)	
	• Social influences (message from chairman acts as social influence)	
	• Environmental context and resources (permanent record is a resource)	
	• Memory, attention and decision processes (attention from second message)	
	Intervention 2	
	PCP	
	• Knowledge (knowledge from guidelines)	
	• Memory, attention and decision processes (attention from EMR)	
	• Social influences (message from chairman acts as social influence)	
	• Environmental context and resources (permanent record is a resource)	
	• Memory, attention and decision processes (attention from second message)	
	• Memory, attention and decision processes (copy of patient letter sent to PCP focuses attention as PCP aware patient may visit for discussion)	
	• Memory, attention and decision processes (attention from patient attending to discuss management options)	
	• Memory, attention and decision processes (decision processes: patient attending to discuss management options)	
	Patient	
	• Knowledge (knowledge from educational materials)	
	• Memory, attention and decision processes (attention from letter to patient to discuss management options with PCP)	
	• Social influences (person sending letter to patient may act as a social influence if this is chairman as for PCPs)	
Davis 2007	Patient	Patient
	• Knowledge (knowledge from osteoporosis information)	• Memory, attention and decision processes (call at 3 months may inadvertently focus patient’s attention rather than simply act as an outcome measurement exercise)
	• Memory, attention and decision processes (S: attention from letter encouraging patient to return to PCP)	
	• Environmental context and resources (S: material resource of letter to take to PCP)	
	• Memory, attention and decision processes (attention from telephone call at 3 months)	
	PCP	
	• Memory, attention and decision processes (S: attention from letter)	
	• Memory, attention and decision processes (S: attention from patient attending for further investigation)	
	• Social influences (S: social influence of orthopaedic surgeon)	
Majumdar 2007	Patient	Patient
	• Knowledge (knowledge from educational materials from Osteoporosis Canada)	• Knowledge (knowledge from educational materials from Osteoporosis Canada)
	• Knowledge (knowledge from one-on-one counselling from case manager)	• Memory, attention and decision processes (attention: patient asked to discuss materials with PCP)
	• Beliefs about consequences (beliefs about consequences of testing and treatment)	• Social influences (social influence of study personnel asking patient to discuss materials with the PCP)
	• Social influences (case manager as social influence for patient to agree to BMD scan and prescription)	PCP
	PCP	• Memory, attention and decision processes (attention from patient attending to discuss the materials)
	• Memory, attention and decision processes (attention from patient attending to discuss the materials)	
	• Environmental context and resources (S: BMD scan is a resource)	
	• Environmental context and resources (T: prescription for bisphosphonates by study physician and dispensed by pharmacy is a resource)	
Solomon 2007	Pharmacists	None identified
	• Knowledge (knowledge of condition)	
	• Knowledge (procedural knowledge of academic detailing)	
	• Skills (skills—practicing physician encounters)	
	• Beliefs about capabilities (beliefs about capabilities targeted using mock scripts)	
	• Goals (reviewed goals of the intervention)	
	• Memory, attention and decision processes (memory/attention—follow-up teleconferences)	
	• Environmental context and resources (provision of logistical support is a resource)	
	PCP	
	• Knowledge (educational visit—knowledge of condition)	
	• Memory, attention and decision processes (decision processes: algorithm for diagnosis and treatment of osteoporosis)	
	• Environmental context and resources (double sided laminated card is a resource)	
	• Environmental context and resources (tear sheet is a resource)	
	• Memory, attention and decision processes (attention from tear sheet)	
	• Environmental context and resources (patient list is a resource)	
	• Memory, attention and decision processes (patient list used during discussion to give examples of patients that should be considered for scan/treatment)	
	• Social influences (pharmacists as social influence)	
	• Environmental context and resources (S: BMD scan offered via automated call is a resource)	
	Patient	
	• Memory, attention and decision processes (S: automated call encouraged members to schedule a BMD scan)	
	• Knowledge (S: from phone call about osteoporosis and risk information)	
	• Beliefs about consequences (S: of condition and testing)	
	• Beliefs about capabilities (S: “only takes 5 min”)	
	• Emotion (S: “painless”, “no need to take off clothes”)	
	• Environmental context and resources (S: resource for scheduling BMD scan)	
	• Memory, attention and decision processes (S: second call offering patient opportunity to schedule BMD scan)	
Cranney 2008	PCP	None identified
	• Knowledge (from two-page educational tool)	
	• Beliefs about consequences (of osteoporosis and benefits/risks of treatment)	
	• Memory, attention and decision processes (attention from letter at 2 weeks post-fracture)	
	• Memory, attention and decision processes (attention from letter at 2 months post-fracture)	
	• Memory, attention and decision processes (treatment algorithm aids decision processes)	
	• Memory, attention and decision processes (attention from patient attending to discuss osteoporosis)	
	• Social influences (endorsement from Osteoporosis Canada acts as social influence)	
	Patient	
	• Memory, attention and decision processes (attention from reminder letter at 2 weeks)	
	• Memory, attention and decision processes (attention from reminder letter at 2 months)	
	• Beliefs about consequences (future fracture risk)	
	• Knowledge (from checklist of risks for fractures and 5-year absolute fracture risk)	
	• Knowledge (from educational booklet about osteoporosis treatment options)	
Majumdar 2008	Patient	Patient
	• Knowledge (of condition from Osteoporosis Canada pamphlet)	• Knowledge (of condition from Osteoporosis Canada pamphlet)
	• Social influences (of Osteoporosis Canada)	• Social influences (of Osteoporosis Canada)
	• Beliefs about consequences (pamphlet highlighting fractures as harbinger of future events)	• Beliefs about consequences (fractures as harbinger of future events)
	• Memory, attention and decision processes (attention: pamphlet emphasising importance of follow-up)	• Memory, attention and decision processes (attention from pamphlet emphasising importance of follow-up)
	• Environmental context and resources (contact information is a resource)	• Environmental context and resources (contact information is a resource)
	• Knowledge (from printed materials with 3 key messages)	• Memory, attention and decision processes (attention from second copy of pamphlet)
	• Knowledge (telephone call reiterated 3 key messages)	PCP
	• Beliefs about consequences (3 key messages addressed beliefs about consequences of investigation/treatment)	• Memory, attention and decision processes (attention from patient attending to discuss pamphlet)
	• Social influences (of nurse during phone call)	
	• Beliefs about consequences (nurse allayed concerns)	
	• Emotions (nurse allayed concerns)	
	• Environmental context and resources (nurse as a resource—answered any questions)	
	PCP	
	• Memory, attention and decision processes (attention from patient attending to discuss management)	
	• Memory, attention and decision processes (attention from patient-specific reminder)	
	• Beliefs about consequences (3 key messages addressed beliefs about consequences of investigation/treatment)	
	• Knowledge (from guidelines)	
	• Social influence (of local opinion leaders)	
	• Environmental context and resources (material resource of printed page with reminder and treatment guidelines forming part of patient’s record)	
Miki 2008	Patient	Patient
	• Knowledge (from 15-min education)	• Knowledge (from 15-min education)
	• Knowledge (education reiterated at follow-up clinic)	PCP
	• Memory, attention and decision processes (T: telephone call/clinic visit to assess adherence may target memory to take medication)	• Memory, attention and decision processes (attention from patient attending for osteoporosis evaluation)
	• Social influences (T: social influence of orthopaedic surgeon to adhere with treatment)	
	PCP	
	• Environmental context and resources (S: evaluation for osteoporosis in hospital including BMD scan is a resource)	
	• Environmental context and resources (T: follow-up in specialised orthopaedic osteoporosis clinic with commencement of treatment as appropriate is a resource)	
	• Environmental context and resources (T: telephone call/clinic visit to monitor adherence and assess for complications is a resource)	
Rozental 2008	PCP	PCP
	• Environmental context and resources (S: BMD scan ordered by surgeon is a resource)	• Knowledge (from guidelines)
	• Memory, attention and decision processes (attention from patient following up with PCP)	• Social influences (of orthopaedic surgeon’s letter)
	Patient	• Social influences (of NOF guidelines)
	• Knowledge (of results of scan)	
	• Memory, attention and decision processes (attention: patient encouraged to follow up with PCP)	
	• Social influences (of encouragement from orthopaedic surgeon to discuss with PCP)	

Notes: PCP = primary care physician. A “T” in front of the code indicates that the code is related solely to osteoporosis treatment with anti-resorptive therapy, and an “S” solely to BMD scanning. The coding specified who the primary recipient of the intervention was, i.e. patient, PCP or pharmacist

Table 2 presents the number of times each of the domains were coded with respect to BMD scanning and osteoporosis treatment with anti-resorptive therapy in the intervention group of each of the studies. The recipient of the intervention, i.e. patient or PCP, was also specified.

**Table 2:** Number of times each domain coded in the intervention group

		Gardner (2005)		Feldstein 1 (2006)	Feldstein 2 (2006)		Davis (2007)		Majumdar (2007)		Solomon (2007)			Cranney (2008)		Majumdar (2008)		Miki (2008)		Rozental (2008)		Total
Domains	Behaviour	Pt.	PCP	PCP	Pt.	PCP	Pt.	PCP	Pt.	PCP	Pt.	PCP	Pharm	Pt.	PCP	Pt.	PCP	Pt.	PCP	Pt.	PCP	
1. Knowledge	Scan	1		1	1	1	1		2		1	1	2	2	1	3	1	2		1		21
	Treatment	1		1	1	1	1		2			1	2	2	1	3	1	2		1		20
2. Skills	Scan												1									1
	Treatment												1									1
3. Social/professional role and identity	Scan																					0
	Treatment																					0
4. Beliefs about capabilities	Scan										1		1									2
	Treatment												1									1
5. Optimism	Scan																					0
	Treatment																					0
6. Beliefs about consequences	Scan	1							1		1			1	1	3	1					9
	Treatment	2							1					1	1	3	1					9
7. Reinforcement	Scan																					
	Treatment																					0
8. Intentions	Scan																					0
	Treatment																					0
9. Goals	Scan		1										1									2
	Treatment		1										1									2
10. Memory, attention and decision processes	Scan	1	2	2	1	5	2	2		1	2	3	1	2	4	1	2			1	1	33
	Treatment	1	2	2	1	5	1			1		3	1	2	4	1	2	1		1	1	29
11. Environmental context and resources	Scan	1		1		1	1			1	1	4	1			2	1		1		1	16
	Treatment	1		1		1				1		3	1			2	1		2			13
12. Social influences	Scan			1	1	1		1	1			1			1	2	1			1		11
	Treatment			1	1	1			1			1			1	2	1	1		1		11
13. Emotion	Scan										1					1						2
	Treatment															1						1
14. Behavioural regulation	Scan																					0
	Treatment																					0
Total no. of domains targeted	Scan	4	3	5	3	8	4	3	4	2	7	9	7	5	7	12	6	2	1	3	2	
	Treatment	5	3	5	3	8	2		4	2		8	7	5	7	12	6	4	2	3	1	

*Pt.* patient, *PCP* primary care physician, *Pharm* pharmacist, *Scan* BMD scan, *Treatment* osteoporosis treatment with anti-resorptive therapy

The domain coded most frequently was “memory, attention and decision processes” (10 of 10 interventions; BMD scanning coded 33 times; treatment coded 29 times). The second most frequently coded domain was “knowledge” (10 of 10 interventions; BMD scanning coded 21 times; treatment coded 20 times), closely followed by “environmental context and resources” (9 of 10 interventions; BMD scanning coded 16 times; treatment coded 13 times), “social influences” (9 of 10 interventions; BMD scanning coded 11 times; treatment coded 11 times) and then “beliefs about consequences” (5 of 10 interventions; BMD scanning coded 9 times; treatment coded 9 times). In all of the studies, there was a combination of at least four of these five domains. There were five domains that were never coded: “social/professional role and identity”; “optimism”; “reinforcement”; “intentions”; and “behavioural regulation”. The remaining four domains (“skills”; “beliefs about capabilities”; “goals”; “emotion”) were coded only once or twice with respect to both BMD scanning and treatment.

Table 3 presents the number of times each of the domains were coded with respect to BMD scanning and osteoporosis treatment with anti-resorptive therapy in the *control groups* of each of the studies.

**Table 3:** Number of times each domain coded in the control group

		Gardner (2005)		Feldstein (2006)		Davis (2007)		Majumdar (2007)		Solomon (2007)			Cranney (2008)		Majumdar (2008)		Miki (2008)		Rozental (2008)		Total
Domains	Behaviour	Pt.	PCP	Pt.	PCP	Pt.	PCP	Pt.	PCP	Pt	PCP	Pharm	Pt.	PCP	Pt.	PCP	Pt.	PCP	Pt.	PCP	
1. Knowledge	Scan							1							1		1			1	4
	Treatment							1							1		1			1	4
2. Skills	Scan																				
	Treatment																				
3. Social/ professional role and identity	Scan																				
	Treatment																				
4. Beliefs about capabilities	Scan																				
	Treatment																				
5. Optimism	Scan																				
	Treatment																				
6. Beliefs about consequences	Scan														1						1
	Treatment														1						1
7. Reinforcement	Scan																				
	Treatment																				
8. Intentions	Scan																				
	Treatment																				
9. Goals	Scan																				
	Treatment																				
10. Memory, attention and decision processes	Scan					1		1	1						2	1		1			7
	Treatment					1		1	1						2	1		1			7
11. Environmental context and resources	Scan														1						1
	Treatment														1						1
12. Social influences	Scan							1							1					2	4
	Treatment							1							1					2	4
13. Emotion	Scan																				
	Treatment																				
14. Behavioural regulation	Scan																				
	Treatment																				
Total no. of domains targeted	Scan	0	0	0	0	1	0	3	1	0	0	0	0	0	6	1	1	1	0	3	
	Treatment	0	0	0	0	1	0	3	1	0	0	0	0	0	6	1	1	1	0	3	

*Pt.* patient, *PCP* primary care physician, *Pharm* pharmacist, *Scan* BMD scan, *Treatment* osteoporosis treatment with anti-resorptive therapy

We identified and coded domains in the control group for only five of the studies. This was due to the poor descriptions of the care given to the control groups. Fewer elements were coded, and these were spread over fewer domains. The domains most frequently identified in the description of the control group were again “memory, attention and decision processes” (4 of 9 control groups; BMD scanning coded 7 times; treatment coded 7 times), “knowledge” (4 of 9 control groups; BMD scanning coded 4 times; treatment coded 4 times) and “social influences” (3 of 9 control groups; BMD scanning coded 4 times; treatment coded 4 times). “Beliefs about consequences” and “environmental context and resources” were coded once each for both BMD scanning and treatment.

Across the studies described as having been tailored to identified barriers and facilitators, each reported lack of clarity regarding which physician was responsible for the investigation and management of osteoporosis following a fragility fracture as a significant barrier but the interventions themselves were not described to target the “social/professional role and identity” domain for any of these studies. The interventions instead addressed this barrier either by taking the behaviour out of the hands of the PCP altogether (“environmental context and resources”) or by using reminders for the PCP to perform the behaviour (“memory, attention and decision processes”).

On the whole, the remainder of the barriers and facilitators that were identified in the studies were reflected in the frequency of the domains coded, with the exception of the “social influences” domain. This was the fourth most frequently coded domain and yet was only referred to in one study as being important in changing health care professionals’ behaviour [41]. Only one study addressed the “social influences” domain, by selecting local opinion leaders that had been identified as educationally influential physician peers in the area of osteoporosis by PCPs through a validated questionnaire prior to the intervention [43].

### Relationship between total number of times the domains are coded within an intervention and effect size

**Fig. 1:**
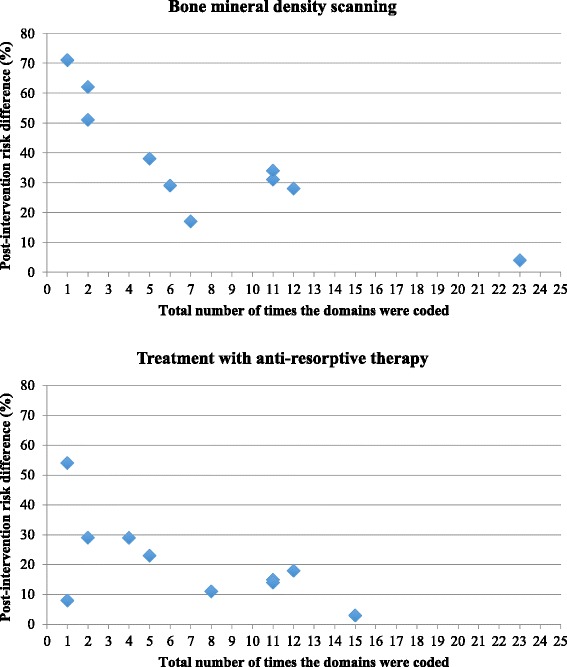
Scatterplot of total number of times the domains were coded and intervention effect size (BMD scanning and treatment with anti-resorptive therapy)

Table 4 summarises the *total* number of times the domains were coded, for each study intervention and control group by BMD scanning and osteoporosis treatment with anti-resorptive therapy. Scatterplots are presented in Fig. 1.

**Table 4:** Total number of times domains coded within intervention and control groups

Studies	Total no. times any domain coded Intervention		Total no. times any domain coded control		Intervention minus control		Post-intervention risk difference (%)	
	Scanning	Treatment	Scanning	Treatment	Scanning	Treatment	Scanning	Treatment
Gardner 2005	7	8	0	0	7	8	17	11
Feldstein 2006								
Intervention 1	5	5	0	0	5	5	38	23
Intervention 2	11	11			11	11	31	15
Davis 2007	7	2	1	1	6	1	29	54
Majumdar 2007	6	6	4	4	2	2	51	29
Solomon 2007	23	15	0	0	23	15	4	3
Cranney 2008	12	12	0	0	12	12	28	18
Majumdar 2008	18	18	7	7	11	11	34	14
Miki 2008	3	6	2	2	1	4	71	29
Rozental 2008	5	4	3	3	2	1	62	8

There was a statistically significant inverse relationship between the total number of times the domains were coded within an intervention and the post-intervention risk difference for BMD scanning, *r* = −0.831, *p* < 0.05 but not for treatment (*r* = −0.615, *p* = 0.058). The sensitivity analysis (not subtracting the number of control group domains) showed similar results (BMD scanning: *r* = −0.704, *p* < 0.05; treatment: *r* = −0.603, *p* = 0.065).

### Relationship between the number of different domains coded within an intervention and the effect size

**Fig. 2:**
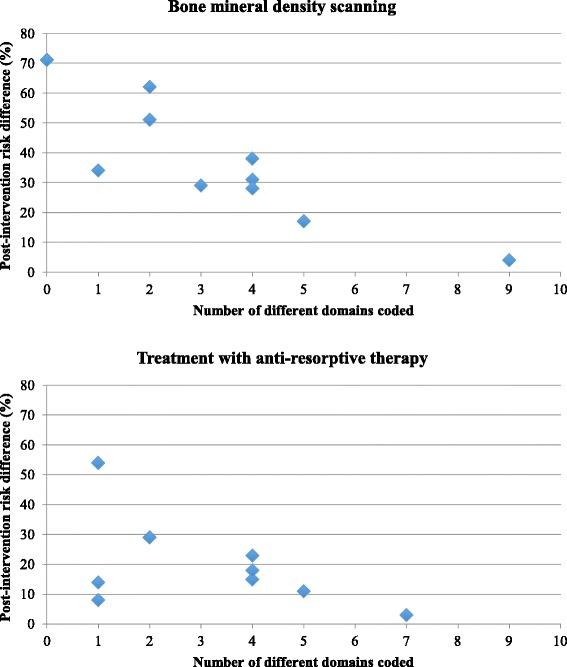
Scatterplot of number of different domains coded and intervention effect size (BMD scanning and treatment with anti-resorptive therapy)

Table 5 summarises the number of *different* domains coded in the intervention and control groups of each of the studies. Scatterplots are presented in Fig. 2.

**Table 5:** Number of different domains coded within intervention and control groups and intervention effect size

Studies	Number of different domains coded Intervention		Number of different domains coded control		Intervention minus control		Post-intervention risk difference (%)	
	Scanning	Treatment	Scanning	Treatment	Scanning	Treatment	Scanning	Treatment
Gardner 2005	5	5	0	0	5	5	17	11
Feldstein 2006								
Intervention 1	4	4	0	0	4	4	38	23
Intervention 2	4	4			4	4	31	15
Davis 2007	4	2	1	1	3	1	29	54
Majumdar 2007	5	5	3	3	2	2	51	29
Solomon 2007	9	7	0	0	9	7	4	3
Cranney 2008	4	4	0	0	4	4	28	18
Majumdar 2008	6	6	5	5	1	1	34	14
Miki 2008	2	4	2	2	0	2	71	29
Rozental 2008	4	3	2	2	2	1	62	8

There was a statistically significant inverse relationship between number of different domains coded within an intervention and the post-intervention risk difference for BMD scanning (*r* = −0.848, *p* < 0.05) but not for treatment (*r* = −0.530, *p* = 0.115). The sensitivity analysis (no subtraction of control group domains) showed similar results for BMD scanning (*r* = −0.728, *p* < 0.05), but for treatment, the relationship became statistically significant (*r* = −0.644, *p* < 0.05).

## Discussion

We conducted a theory-based analysis of the interventions from nine randomised controlled trials (RCTs) included in a systematic review to assess the effectiveness of a variety of interventions to improve the investigation and management of osteoporosis following fragility fracture. Although there are many examples of the prospective use of the TDF in implementation research, to our knowledge, this is the first time that the TDF has been used as a coding framework to retrospectively analyse the factors targeted by implementation interventions in a systematic review.

The theory-based analysis using the TDF highlighted five key domains that appeared to be targeted most frequently by the interventions: “Memory, attention and decision processes”, “knowledge”, “environmental context and resources”, “social influences” and “beliefs about consequences”. For each of the ten interventions from the nine studies, we coded a combination of at least four of these five domains. The results of the exploratory quantitative analysis suggested an inverse relationship between both the number of times the domains were coded and the number of different domains coded and the effect size of the intervention for BMD scanning, but the relationship was less clear with regards to treatment with anti-resorptive therapy.

### Frequently coded domains

“Memory, attention and decision processes” was by far the most frequently coded domain. This suggests that the study authors believed that PCPs need reminders, prompts or decision aids to enable them to perform the behaviours, indicating that they believed that PCPs were either forgetting to perform the behaviour or had impaired decision processes with regard to performing the behaviour. This also addressed the identified barrier of PCPs failing to make the connection between a fracture and osteoporosis.

“Knowledge” was the second most frequently coded domain. For all the interventions that included the patient as a recipient, the “knowledge” domain was coded with respect to the patient. This indicates that the authors believed that in order to ensure that the patient could influence the PCP’s behaviour, they first required a certain amount of knowledge about the investigation and management of osteoporosis following fragility fracture. The use of “knowledge” with regards to the PCP was less consistent (five out of ten interventions) which suggests that the study authors differed in their views about PCPs’ existing knowledge of the condition and its management. This domain encompasses the barriers of a lack of awareness by patients and physicians of the treatment guidelines and efficacy of medications for osteoporosis following fragility fracture.

“Environmental context and resources” was the third most frequently coded domain. It was interesting that in the three studies with the greatest effect sizes for BMD scanning (Miki [44], Rozental [45] and Majumdar [40], with post-intervention risk differences of 71, 62 and 51 %, respectively), a large part of the intervention was coded within this domain. In the Miki study, the BMD scan was ordered by the orthopaedic surgeon and carried out whilst the patient was still in hospital. In Rozental the BMD scan was ordered by the orthopaedic surgeon during the first outpatient clinic visit; and in Majumdar, a case manager arranged the BMD scan. This was coded as a resource, but one could argue that rather than changing the behaviour of the PCP, the effectiveness of the interventions stemmed from the fact that the behaviour was taken completely out of the hands of the PCP and was actually a service delivery change—someone else performed the behaviour instead. In these three examples, the barrier of inadequate access to BMD scanning was addressed. It is worth noting that this domain was also coded for studies with smaller effect sizes for BMD scanning, including Solomon which had the smallest effect size (4 %) [41], and in which it was coded once for the patient, four times for the PCP and once for the pharmacist. This highlights a key point when using the TDF: it is not just *which* domains you target but *how* they are targeted that is important. Just because one targets “environmental context and resources” or “Memory, attention and decision processes’, it does not mean that it is done appropriately or effectively. However, the advantage of using the TDF is that it allows us to use a common language for describing those targets.

Finally, “Beliefs about consequences” was coded fifth most frequently. Interestingly, patient “beliefs about consequences” was targeted in all of the five studies for which this domain was coded but PCP “beliefs about consequences” was only targeted in two of them. This implies that the authors believed that patients had not considered or did not understand the consequences of failing to investigate for and treat osteoporosis post-fracture, and by remedying this, they would be able to change the behaviour of the PCP by, for example, using patient prompts. This addressed barriers such as concerns about whether bisphosphonate treatment might impair fracture-healing, concerns about adverse effects of medications or lack of awareness of the efficacy of medications following fracture.

### Description of intervention and control groups

In addition to the poor reporting of the rationale for the interventions, from some of the descriptions given, it was difficult to extract sufficient detail to be confident that the interventions were being described in a way that would make them replicable. It was also difficult to disentangle what the investigators felt was the content of their intervention (the active ingredients; cf. [47]) from the method that they chose to deliver it (e.g. printed educational materials). Such distinction is important in order to promote greater clarity in the description of interventions [48].

The descriptions of the control groups were often underreported, and in some cases completely absent. This impacted on our ability to code certain domains with confidence. This is a consistent finding throughout the behaviour change literature. Reviews of nearly 1000 behaviour change outcome studies [49–52] found that detailed descriptions of interventions were present in only 5 to 30 %. Failing to report the actual behaviour targeted for change by the intervention or describing the content of the intervention in sufficient detail makes it impossible to identify why an intervention did or did not work [53, 54]. This prevents the replication of successful interventions in wider settings; the introduction of the Template for Intervention Description and Replication checklist (TIDieR) [55] may improve the current situation.

### Relationship between the number of domains targeted and the effect size

The exploratory analysis showed a statistically significant inverse relationship between the total number of *times* the domains were coded and the number of *different* domains coded and the effect size of the intervention for BMD scanning. However, the results for the equivalent analysis on anti-resorptive therapy were not statistically significant. This may be due to the fact that anti-resorptive therapy is itself partially dependent upon BMD scanning. We have shown that the study authors rarely documented the management guideline they were using, but in the majority of cases, the patient needed to have a BMD scan before treatment was commenced. Following the BMD scan, the patient may not have required treatment for osteoporosis so although the patient would have received appropriate care, it would appear as if the intervention had not been successful as the patient was not given subsequent anti-resorptive treatment.

Nevertheless, the inverse relationship demonstrated for BMD scanning was unexpected. We assumed that multifaceted interventions would prove to be associated with larger effect sizes. There may be a range of reasons that explain the inverse relationship, including that it is a chance finding given the small number of studies included in the analysis. Equally, it may be that targeting more domains may not be inherently better; what is likely more important may be to better match the interventions to the domains shown to be relevant for the behaviour, context and population under study [16].

### Limitations and challenges

None of the studies employed the explicit use of theory, which meant that our coding was based on inference from the text. This is not unusual; studies in this area rarely employ theories of behaviour change, or if they do, they fail to report it [56].

The main limitation with the exploratory quantitative analysis of the relationship between domains coded and effect size of the intervention was that there were only 10 data points; outliers had a large influence on the results. Although there was only moderate agreement achieved (*K* = 0.507) following the initial coding, the multidisciplinary coding approach likely allowed for better sensitivity of both the clinical and theoretical content.

There are also challenges to using the TDF itself in this context. Not all domains are necessarily mutually exclusive, with some sharing certain constructs, for example, “action planning” is a part of both “goals” and “behavioural regulation”. Separating “intentions” and “goals” domains was sometimes a challenge. Given the inferential nature of the coding, there were instances when agreeing upon a target domain proved challenging (reflected in our inter-rater reliability). This was in line with previous research in the context of interview studies [57], perhaps reflecting the difficulty of clarifying the boundaries between some domains when using the TDF as a coding framework.

A final challenge involved coding interventions that included a service delivery change. In three studies, the behaviour of performing the BMD scan was taken out of the hands of the target PCP altogether and performed by another individual. We coded such instances as targeting “environmental context and resources” domain. However, the target of this part of the intervention was at an organisational level rather than an individual PCP or patient level. Nevertheless, someone’s behaviour higher in the organisation needed to change for this service delivery to take effect; the TDF could potentially be applied to describe their behaviour as well in future studies if descriptions in intervention reports provide such detail.

## Conclusion

It is possible to use the TDF to retrospectively identify domains targeted by implementation interventions within systematic reviews. Even when the interventions themselves were not explicitly theory-based, the findings suggest that it is possible to attempt to make explicit the implicit theories that formed the basis for intervention. We identified five key domains that appeared to be most frequently targeted by the interventions. Interventions could be optimised by assessing whether these domains appear to be determinants of the target behaviour. We also identified a number of domains that had not been targeted, which could be considered as targets in future interventions if indicated to be barriers or facilitators. Unexpectedly, the results suggested that the effect size was inversely related to the number of domains targeted by an intervention, with the potential implication that focussing on less domains makes interventions more effective. The method proposed may serve as a basis for using the TDF as a means of better understanding the targeted factors in reviews of implementation interventions in other contexts when such factors are not explicitly identified within an existing theoretical framework.

## Additional files

Additional file 1:
**Data extraction form for theory-based analysis.**
Additional file 2:
**Detailed coding of domains targeted in the intervention and control groups.**
