# Supplementary material for: Understanding effects in reviews of implementation interventions using the Theoretical Domains Framework
Source: Implement Sci. 2015 Jun 17;10:90. doi: 10.1186/s13012-015-0280-7 (PMC4469259; doi:10.1186/s13012-015-0280-7)
Supplement: Additional file 1: — Data extraction form for theory-based analysis. [file 13012_2015_280_MOESM1_ESM.docx]

Additional File 1: Data extraction form for theory based analysis.

|  |  |  |  | **Domains** | **Targeted** |  |  |
| --- | --- | --- | --- | --- | --- | --- | --- |
| **Study** | **Description of Intervention** | **Description of Control Group** | **BMD scan (I)** | **BMD scan (C)** | **Treatment with bisphos. (I)** | **Treatment with bisphos. (C)** | **Comments** |
| Gardner 2005 | **Content**  15 minutes patient education; five questions to take to PCP regarding  investigation, diagnosis and management of osteoporosis; patient reminder at six weeks post-op.  **Delivery**  Visit by clinical research  coordinator during hospitalization; printed copy of questions; phone call. | Prior to discharge, patients given two page  pamphlet on fall prevention based on a  National Osteoporosis  Foundation publication. |  |  |  |  |  |
| Feldstein 2006 | **Intervention one:**  **Content**  Physician alert and education.  **Delivery**  Patient-specific electronic medical record (EMR) in-basket message from chairman of the osteoporosis quality improvement committee; internal and external guideline resources; second message sent at three months if no investigation/treatment carried out.  **Intervention two:**  **Content**  Physician alert and education; patient reminder and education  copied to PCP.  **Delivery**  Patient-specific EMR in-basket  message as above with copy of patient reminder; printed educational materials in advisory letter to patient. | Usual care - if patient is  hospitalized for a fracture, the PCP receives a copy of the discharge  summary and the patient  is followed-up by  orthopaedists in a fracture clinic. |  |  |  |  |  |
| Davis 2007 | **Content**  Patient education and advice to visit PCP for further investigation; physician alert.  **Delivery**  Osteoporosis information; letter for patient to take to PCP from orthopaedic surgeon. | Usual care for the fracture and a phone call at three months (general health inquiry) and 6 months to determine whether osteoporosis investigation and treatment had occurred. |  |  |  |  |  |
| Majumdar 2007 | **Content**  Usual care; patient education; outpatient BMD test; prescription for bisphosphonates for patients with low bone mass; communication to PCPs regarding results and treatment plans.  **Delivery**  Case-manager – provided one-on-one counselling; arranged BMD test; obtained prescription from study physician to be dispensed by local community pharmacy. | Study personnel provided  counselling about fall  prevention and intake of  calcium and vitamin D;  educational materials  from osteoporosis Canada provided and patients asked to discuss the material with their  PCP. |  |  |  |  |  |
| Solomon 2007 | **Content**  Physician education; physician alert; patient education; patient  invitation to attend BMD scan.  **Delivery**  One-on-one educational visit with PCP conducted by specially trained pharmacists who work  with HBCBSNJ as physician educators; continuing medical  education (CME) program; list of at-risk patients given to PCP and discussed at meeting; printed  educational materials and letter from HBCBSNJ to patient; automated phone call invitation for BMD scan. | No description, assumed  usual care. |  |  |  |  |  |
| Cranney 2008 | **Content**  Physician alert; physician education; patient reminder recommending F/U visit with PCP; patient education.  **Delivery**  Personalised letter mailed to PCP by research coordinator at two weeks and two months post-fracture; two page educational tool and treatment algorithm from Osteoporosis Canada**’**s clinical practice guidelines; mailed patient reminder letter at two weeks and two months post-fracture; educational booklet. | Usual care. Patients and  PCPs were not sent any  communication until trial  completed. |  |  |  |  |  |
| Majumdar 2008 | **Content**  Patient education and advice to discuss osteoporosis with PCP;  patient-specific reminders to PCPs; physician education.  **Delivery**  Phone counselling session to patients by experienced registered  nurse; physician reminder sent by fax or mail; evidence based treatment guidelines endorsed by opinion leaders sent to PCPs. | Given Osteoporosis  Canada pamphlet and  encouraged to discuss with PCP, second copy  mailed to patient. PCPs  routinely notified that  their patients had been  treated for a wrist fracture and informed of F/U plans and appointment. |  |  |  |  |  |
| Miki 2008 | **Content**  Patient education; osteoporosis evaluation; calcium and vitamin D commenced; patient review and bisphosphonate commenced as appropriate; monitoring of adherence to medication and  complications; transfer of responsibility for medication adherence and patient management to PCP after six months.  **Delivery**  15 minutes education to patient and families whilst in hospital from one of the investigators; inpatient blood tests and BMD scan; F/U  outpatient orthopaedic clinic appointment between two weeks and one month post-op; phone call to patient or clinic visit at two and six months. | 15 minutes education on hip fractures, fracture prevention and osteoporosis from one of  the investigators; advised  to see PCP for osteoporosis evaluation;  commenced on calcium  and vitamin D. |  |  |  |  |  |
| Rozental 2008 | Intervention one:  **Content**  BMD scan with results forwarded to PCP.  **Delivery**  Scan ordered by orthopaedic surgeon during patient**’**s initial  office visit for fracture care; results forwarded by mail and email to PCP; results discussed with patient and patient encouraged to follow up with PCP. | Intervention two:  Letter sent by email and  mail to PCP outlining  national guidelines for  evaluating and treating  osteoporosis after fragility fracture; the guidelines included ordering a BMD scan within six months of injury. |  |  |  |  |  |
