# Supplementary material for: Understanding effects in reviews of implementation interventions using the Theoretical Domains Framework
Source: Implement Sci. 2015 Jun 17;10:90. doi: 10.1186/s13012-015-0280-7 (PMC4469259; doi:10.1186/s13012-015-0280-7)
Supplement: Additional file 2: — Detailed coding of domains targeted in the intervention and control groups. [file 13012_2015_280_MOESM2_ESM.docx]

Additional File 2: Detailed coding of domains targeted in the intervention and control groups.

| **Study** | **Description of Intervention** | **Domains targeted (by recipient)** | **Description of Control** | **Domains targeted** |
| --- | --- | --- | --- | --- |
| Gardner 2005 | Prior to discharge, patients received a *fifteen-minute visit by a clinical research coordinator* who had been trained regarding the discussion of the *role of osteoporosis in hip fractures, the importance of preventing future fractures*, and the *effectiveness of currently available therapies*. Instead of a fall-prevention brochure, patients were given a *printed copy of five questions* to bring to their PCP after discharge. The five questions were as follows:  1. When are you going to address my osteoporosis?  2. What kind of osteoporosis do I have, and how bad is it?  3. When are you going to perform a DEXA scan?  4. When are you going to give me calcium, vitamin D, exercise, and fall prevention?  5. What drugs are you going to prescribe to treat my osteoporosis?  *Patients were contacted by telephone* at six weeks postoperatively and *reminded about the importance of follow-up with their PCP and the need for management of the osteoporosis*. Messages were not left; patients were considered to have been contacted only if they were spoken to directly. Patients were mailed another copy of the questions for their PCP if necessary. | Patient  1 (knowledge from 15 min educational visit)  T 6 (effectiveness of therapies)  11 (material resource of questions)  10 (attention from telephone call at 6 weeks)  6 (call may have targeted beliefs about consequences of seeking follow up with PCP)  PCP  9 (questions are goals and action plans)  10 (questions focus attention)  10 (attention from patient attending to discuss management of osteoporosis) | Prior to discharge, patients given a two-page pamphlet on fall prevention, entitled  “Creating a Fall Proof Home,” based on a National Osteoporosis Foundation publication. The pamphlet outlined home safety tips and mentioned osteoporosis once in the introduction as follows: “osteoporosis can increase your chances of experiencing fractures of the hip, wrist or vertebrae,” but it did not expand on the topic. | None identified. |
| Feldstein 2006 | **Intervention one:**  PCPs received *patient-specific electronic medical record (EMR) in-basket messages* for their enrolled patients from the *chairman of the osteoporosis quality-improvement committee*. These in-basket messages are an EMR-based e-mail communication used exclusively for patient care activities. The letter-style message *informed the provider of the patient’s risk of osteoporosis* based upon the patient’s age and prior fracture and stated *the need for evaluation and treatment.* The message listed *internal and external guideline resources* that provided detailed recommendations regarding evaluation, calcium and vitamin D intake, lifestyle, and medication. The PCP was also given the option of contacting the sender of the message for additional information.  When an in-basket message is selected, a clinician is automatically in the patient’s medical record and can link to the patient’s history, order a procedure or medication, or send a message to request a patient contact. The *EMR reminder created a permanent record*; it was accessible in the patient’s medical record at future visits and could be acted upon at a later date. At 3 months after the first message, *a second patient-specific message* was sent to PCPs who had not ordered a BMD or pharmacological osteoporosis treatment for enrolled patients.  **Intervention two:**  As above, with the addition of:  Patients received a single mailing of an *advisory letter with educational materials*. The letter was similar in content to the one received by the PCP; it identified the patient’s risk, discussed clinical guideline recommendations, and *requested that the patient discuss management options with her PCP*. Enclosed in the mailing were educational materials addressing menopause, osteoporosis, calcium and vitamin D, physical activity, home safety, and fall prevention. *PCPs assigned to this study arm received a copy of the letter sent to the patient* when the PCP received the in-basket reminder. | **Intervention 1**  PCP  1 (knowledge from guidelines)  10 (attention from EMR)  12 (message from chairman acts as social influence)  11 (permanent record is a resource)  10 (attention from second message)  **Intervention 2**  PCP  1 (knowledge from guidelines)  10 (attention from EMR)  12 (message from chairman acts as social influence)  11 (permanent record is a resource)  10 (attention from second message)  10 (copy of patient letter sent to PCP focuses attention as PCP aware patient may visit for discussion)  10 (attention from patient attending to discuss management options)  10 (decision processes: patient attending to discuss management options)  Patient  1 (knowledge from educational materials)  10 (attention from letter to patient to discuss management options with PCP)  12 (person sending letter to patient may act as a social influence if this is chairman as for PCPs) | Patients in usual care continued to receive care at the HMO through their normal care processes. Most patients select a PCP and may seek care from that clinician at a single site, although they are free to seek care through other clinicians and in other settings, such as urgent care. At the time of the study, patients were not required to select a PCP. Generally, if a patient is hospitalised for a fracture, the PCP receives a copy of the discharge summary. When fractures are evaluated in the outpatient setting, they are most frequently seen in the emergency department or minor trauma clinic, with follow-up provided by orthopaedists in a fracture clinic. The PCP may evaluate outpatient fractures, but this is less likely. | None identified. |
| Davis 2007 | The Intervention consisted of usual care plus three elements, specifically: (i) usual care for the fracture including surgical treatment, (ii) *osteoporosis information* and a *letter for participants that encouraged them to return to their PCPs for further investigation*, (iii) *a request for participants to take a letter from the orthopedic surgeon to the PCP alerting that physician to the hip fracture and encouraging osteoporosis investigation*, and (iv) a *telephone call at 3 months* and 6 months to determine whether osteoporosis investigation and treatment had occurred. | Patient  1 (knowledge from osteoporosis information)  S 10 (attention from letter encouraging patient to return to PCP)  S 11 (material resource of letter to take to PCP)  10 (attention from telephone call at 3 months)  PCP  S 10 (attention from letter)  S 10 (attention from patient attending for further investigation)  S 12 (social influence of orthopaedic surgeon) | Usual care consisted of: (i) usual care for the fracture including surgical treatment, and (ii) a *telephone call at 3 months* (general health inquiry) and 6 months to determine whether osteoporosis investigation and treatment had occurred. | Patient  10 (call at 3 months may inadvertently focus patient’s attention rather than simply act as an outcome measurement exercise) |
| Majumdar 2007 | In addition to *usual care*, the study case manager provided additional  *one-on-one counseling* about the importance of BMD testing and the *ability of bisphosphonate therapy and other treatments to reduce the risk of future fracture*. Furthermore, the case manager arranged for an *outpatient BMD test* as soon as the patient had convalesced and returned to the community.  BMD test wait times in Capital Health are less than 1 week. Based on results of the BMD test, the case manager discussed risks and benefits of bisphosphonate therapy and *arranged for local community pharmacies to dispense prescriptions written by a study physician* for alendronate, 70 mg/wk, or risedronate, 35 mg/wk, for patients with low bone mass who wanted to start pharmacotherapy. The goal was to have BMD testing and start of medication completed in the 12 weeks after hip fracture. This was done to ensure that only patients with low bone mass received treatment (vs. starting bisphosphonate therapy during hospitalization in all patients with hip fracture) and to offset concerns about the potential for bisphosphonate therapy to impair healing and outcomes related to surgical fixation. All results and treatment plans were communicated to the PCP of record. | Patient  1 (knowledge from educational materials from Osteoporosis Canada)  1 (knowledge from one-on-one counselling from case manager)  6 (beliefs about consequences of testing and treatment)  12 (case manager as social influence for patient to agree to BMD scan and prescription)    PCP  10 (attention from patient attending to discuss the materials)  S 11 (BMD scan is a resource)  T 11 (prescription for bisphosphonates by study physician and dispensed by pharmacy is a resource) | Patients received more education and study related attention than true usual care as practiced in most Canadian or US centers. Study personnel provided counseling about fall prevention and the need for additional intake of calcium and vitamin D. In addition, study personnel provided *educational materials from Osteoporosis Canada* (Toronto, Ontario) and *asked patients and caregivers to discuss this material with their PCP*. | Patient  1 (knowledge from educational materials from Osteoporosis Canada)  10 (attention: patient asked to discuss materials with PCP)  12 (social influence of study personnel asking patient to discuss materials with the PCP)  PCP  10 (attention from patient attending to discuss the materials) |
| Solomon 2007 | The main intervention consisted of *one-on-one educational visits with PCPs*. The visits were conducted by *specially trained pharmacists* who work with HBCBSNJ as physician educators. These pharmacists also underwent a *1-day training program focused on osteoporosis* and conducted by 2 of the study authors. This program inclu*ded lectures on the epidemiology, diagnosis, and treatment of osteoporosis*. Also, it reviewed *principles of academic detailing and the specific goals of this intervention*. *Mock scripts* were used for *practicing physician encounters*, and several *follow-up teleconferences* were conducted to review materials, practice educational encounters, and provide logistical support to the educators. We developed a *continuing medical education (CME) program* (accredited by Harvard Medical School**’**s CME department) that was distributed in the setting of the physician visit. The materials consisted of *brief summaries of osteoporosis epidemiology, diagnosis, and treatment*. Also, we provided doctors with an *algorithm for diagnosis and treatment of osteoporosis and a guide to osteoporosis pharmacotherapy*. A version of this material was reproduced on 1 *double-sided laminated card* small enough to fit into a coat pocket. The educators also offered the doctors and their staff ***“****tear sheets****”*** for patients that resembled prescription pads with check boxes for fall prevention, calcium and vitamin D use, bone mineral density testing, and treatment. We supplied patient materials on fall prevention to the PCP**’**s office staff. In addition, the study paid for doctors to apply for CME credit if they completed a post-visit test. Each PCP in the intervention groups received a *list of her HBCBSNJ patients at-risk for osteoporosis*. The *educators used this list during the one-on-one visit with doctors to give examples of patients that should be considered for BMD testing and/or treatment*. Patients received an *introductory letter from HBCBSNJ and then an automated telephone call from HBCBSNJ inviting them to undergo BMD testing*. This call employed interactive voice response technology that has been used for other screening tests. An English-speaking, female voice was used for all calls. If the IVR system did not reach the intended member, a message stated that HBCBSNJ was calling to follow up on a recent letter and invited the member to call a toll-free number. Calls were attempted at different times during the day to enhance success. A successful contact reached the intended member. A failed contact was defined as 1 of the following: answering machine; live person, but not the intended member; wrong/disconnected number; busy signal; no answer; or hanging up. When a member was contacted, an interactive script began. The *script included risk information, encouraged members to schedule a BMD test, and praised prevention behaviours*. Examples from the script include "Osteoporosis is a disease that makes your bones weak and likely to break easily. Because osteoporosis is a painless disease, you can have osteoporosis and not be aware of it" and "The best way to tell if a person is at risk for osteoporosis is to have a bone density test. The test only takes about 5 minutes, you don't have to take off your clothes, and it's painless." The script did not mention any specific medication or treatment for osteoporosis. Each HBCBSNJ member was offered the *opportunity to schedule a BMD test*. If a member said yes, he or she was transferred to the radiology scheduling service. If the member said no, he or she was asked questions regarding barriers to testing (e.g., cost, other health priorities). Two weeks after the first call, the IVR system initiated a second call. To respect members' privacy, the IVR system called only those members who (1) had not been reached or (2) did not transfer to schedule a BMD test during the first call but otherwise answered questions. This *second call again offered the opportunity to schedule a BMD test.* | Pharmacists  1 (knowledge of condition)  1 (procedural knowledge of academic detailing)  2 (skills – practicing physician encounters)  4 (beliefs about capabilities targeted using mock scripts)  9 (reviewed goals of the intervention)  10 (memory/attention – follow-up teleconferences)  11 (provision of logistical support is a resource)  PCP  1 (educational visit – knowledge of condition)  10 (decision processes: algorithm for diagnosis and treatment of osteoporosis)  11 (double sided laminated card is a resource)  11 (tear sheet is a resource)  10 (attention from tear sheet)  11 (patient list is a resource)  10 (patient list used during discussion to give examples of patients that should be considered for scan/treatment)  12 (pharmacists as social influence)  S 11 (BMD scan offered via automated call is a resource)  Patient  S 10 (automated call encouraged members to schedule a BMD scan)  S 1 (knowledge from phone call about osteoporosis and risk information)  S 6 (beliefs about consequences of condition and testing)  S 4 (beliefs about capabilities – ‘only takes 5 mins’)  S 13 (emotion – ‘painless’, ‘no need to take off clothes’)  S 11 (resource for scheduling BMD scan)  S 10 (second call offering patient opportunity to schedule BMD scan) | No description, assumed usual care. | None identified. |
| Cranney 2008 | The intervention was directed at both the patient and physician.  Physician component: The research coordinator *mailed a letter to the PCPs at 2 weeks and 2 months post fracture*. The personalized letter notified the PCP that their patient had a recent wrist fracture and highlighted that wrist fractures can be associated with osteoporosis, and that assessment for osteoporosis treatment is recommended for women with wrist fractures. A *two-page educational tool* that included a summary of recommended osteoporosis therapies for patients with fractures, including benefits and risks of these medications based on the results of systematic reviews. *A treatment algorithm* for patients with fragility fractures *from Osteoporosis Canada****’****s* *clinical practice guidelines* was included.  Patient component: A *reminder letter was mailed to the women at 2 weeks and 2 months after their fracture*. The letter *recommended they schedule a follow-up visit with their PCP to discuss osteoporosis*. A *one-page checklist of risks for fractures was included*, so the woman could calculate her five-year absolute fracture risk *to discuss with her PCP, during their visit*. An *educational booklet*, which included information about osteoporosis, an evidence-based summary of treatment options for osteoporosis, and recommended lifestyle changes was provided. | PCP  1 (knowledge from two-page educational tool)  6 (beliefs about consequences of osteoporosis and benefits/risks of treatment)  10 (attention from letter at 2 weeks post-fracture)  10 (attention from letter at 2 months post-fracture)  10 (treatment algorithm aids decision processes)  10 (attention from patient attending to discuss osteoporosis)  12 (endorsement from Osteoporosis Canada acts as social influence)  Patient  10 (attention from reminder letter at 2 weeks)  10 (attention from reminder letter at 2 months)  6 (beliefs about consequences – future fracture risk)  1 (knowledge from checklist of risks for fractures and five year absolute fracture risk)  1 (knowledge from educational booklet about osteoporosis treatment options) | Patients and PCPs were not sent any communication until the trial was completed, at which point they were sent the educational material. | None identified. |
| Majumdar 2008 | Each patient received a package that included instructions for cast care, information about the study and *an Osteoporosis Canada pamphlet entitled “Osteoporosis: Are You at Risk?” T*he pamphlet described osteoporosis and its risk factors (*highlighting the importance of fractures as a harbinger of future events), diagnosis and treatments*; emphasized the importance of follow-up; and *provided contact information* (in the form of a toll-free number and a website address).  The overall purpose of the intervention was to *deliver the following 3 messages to PCPs: (1) this patient is (you are) at high risk of osteoporosis, and a BMD test is needed; (2) without treatment, this patient (you) may be at risk of a future fracture sooner rather than later (i.e., within 1 year); and (3) bisphosphonate treatment for fracture patients with low bone mass reduces the risk of future fracture by up to 50%*. The third of these messages was qualified, however, by stating that bisphosphonates are not appropriate for all patients and that acceptable treatment alternatives include calcitonin (administered intranasally), hormone replacement therapy and raloxifene.  The intervention consisted of 3 components. First, we provided a *brief counseling session to patients by telephone, reiterating the same messages as the print materials*. This counseling was provided by an experienced registered nurse who had additional training and expertise in the diagnosis and treatment of osteoporosis. Beyond delivering the 3 study messages, the *nurse answered questions and allayed concerns while emphasizing the importance of the patient communicating with his or her PCP*. The nurse did not interact directly with PCPs. Second, we sent (*by fax or mail) a patient-specific reminder to each PCP notifying the PCP that his or her patient had recently received treatment for a wrist fracture and that the occurrence of the fracture indicated that the patient was at risk for osteoporosis*. Third, *evidence-based treatment guidelines, representing an actionable summary of available osteoporosis guidelines and having endorsement from 5 local opinion leaders, were sent to these PCP*s. The local opinion leaders were nominated by regional PCPs who had received a previously validated sociometric questionnaire designed to identify educationally influential physician peers in the area of osteoporosis. The *reminder and the treatment guideline were designed to fit together on one printed page,* were *signed by the opinion leaders* and were intended to become *part of the patient’s medical record*. | Patient  1 (knowledge of condition from Osteoporosis Canada pamphlet)  12 (social influence of Osteoporosis Canada)  6 (beliefs about consequences –pamphlet highlighting fractures as harbinger of future events)  10 (attention: pamphlet emphasising importance of follow up)  11 (contact information is a resource)  1 (knowledge from printed materials with 3 key messages)  1 (telephone call reiterated 3 key messages)  6 (3 key messages addressed beliefs about consequences of investigation/treatment)  12 (social influence of nurse during phone call)  6 (beliefs about consequences – nurse allayed concerns)  13 (emotions – nurse allayed concerns)  11 (nurse as a resource – answered any questions)  PCP  10 (attention from patient attending to discuss management)  10 (attention from patient-specific reminder)  6 (3 key messages addressed beliefs about consequences of investigation/treatment)  1 (knowledge from guidelines)  12 (social influence of local opinion leaders)  11 (material resource of printed page with reminder and treatment guidelines forming part of patient’s record) | Each patient received a package that included instructions for cast care, information about the study and an *Osteoporosis Canada pamphlet entitled “Osteoporosis: Are You at Risk?”* The pamphlet described osteoporosis and its risk factors (*highlighting the importance*  *of fractures as a harbinger of future events)*, *diagnosis and treatments*; emphasized the importance of follow-up;  and *provided contact information* (in the form of a toll-free number and a website address). We *mailed another copy of the Osteoporosis Canada pamphlet to patients, encouraging them to read it and discuss it with their respective PCP*. PCPs were routinely notified that their patients had been treated for a wrist fracture in the emergency department and were informed of follow-up plans and appointments. | Patient  1 (knowledge of condition from Osteoporosis Canada pamphlet)  12 (social influence of Osteoporosis Canada)  6 (beliefs about consequences – fractures as harbinger of future events)  10 (attention from pamphlet emphasising importance of follow up)  11 (contact information is a resource)  10 (attention from second copy of pamphlet)  PCP  10 (attention from patient attending to discuss pamphlet) |
| Miki 2008 | *Patients and families received fifteen minutes of education on hip fractures, fracture prevention, and osteoporosis* from one of the investigators.  Patients received an *osteoporosis evaluation in the hospital*. The workup entailed assessment of *bone density of the lumbar spine and unaffected hip by a dual x-ray absorptiometry scan* and evaluation of serum measures of parathyroid hormone, 25-hydroxyvitamin D, white blood-cell count, hemoglobin, hematocrit, platelet count, sodium, potassium, chloride, bicarbonate, blood urea nitrogen, creatinine, calcium, magnesium, and phosphate. Luteinizing hormone levels were also screened in male subjects. The dual x-ray absorptiometry scan and blood studies were typically obtained on the first or second postoperative day. Prior to discharge, patients in the intervention group began taking 1500 mg of calcium and 800 IU of vitamin D3 daily and were given a *follow-up appointment between two weeks and one month postoperatively in a specialized orthopaedic osteoporosis clinic*. The clinic was run by the senior author, a practicing orthopaedic surgeon. At the *follow-up appointment*, the investigative team *reviewed the results of the laboratory studies and dual x-ray absorptiometry, reinforced education, and started treatment with 35 mg of risedronate.* The patients were followed at *two months and six months postoperatively with clinic visits or telephone calls to determine adherence with the medication and to monitor for complications*. The responsibility for medication adherence and management of the patient after six months was transferred to the PCP. | Patient  1 (knowledge from 15 minutes education)  1 (education reiterated at follow up clinic)  T 10 (telephone call/clinic visit to assess adherence may target memory to take medication)  T 12 (social influence of orthopaedic surgeon to adhere with treatment)  PCP  S 11 (evaluation for osteoporosis in hospital including BMD scan is a resource)  T 11 (follow up in specialised orthopaedic osteoporosis clinic with commencement of treatment as appropriate is a resource)  T 11 (telephone call/clinic visit to monitor adherence and assess for complications is a resource) | *Patients and families received fifteen minutes of education on hip fractures, fracture prevention, and osteoporosis* from one of the investigators.  Patients began taking 1500 mg of calcium and 800 IU of vitamin D3 daily. Prior to discharge, *patients and their families were*  *instructed to approach their PCPs for an*  *osteoporosis evaluation.* A non-blinded follow-up telephone call was made at six months to assess the workup and treatment  of osteoporosis for each patient. If a workup or therapy had not been initiated, patients were once again advised to seek care from their PCP and were also invited to visit the orthopaedic osteoporosis clinic. | Patient  1 (knowledge from 15 minutes education)  PCP  10 (attention from patient attending for osteoporosis evaluation) |
| Rozental 2008 | Intervention one:  The treating *orthopaedic surgeon ordered a BMD test* during the patient’s initial office visit for fracture care. The *results were reviewed and forwarded (by mail and e-mail) to the PCP of record.*  *Results discussed with patient and patient encouraged to follow up with PCP* (correspondence with author). | PCP  S 11 (BMD scan ordered by surgeon is a resource)  10 (attention from patient following up with PCP)  Patient  1 (knowledge of results of scan)  10 (attention: patient encouraged to follow up with PCP)  12 (social influence of encouragement from orthopaedic surgeon to discuss with PCP) | Intervention two:  The treating *orthopaedic surgeon sent a brief letter (by mail and e-mail) to the patient’s PCP outlining national guidelines for evaluating and treating osteoporosis after fragility fractures.* These guidelines included ordering a BMD examination within six months after the injury. Final treatment decisions were made by the PCP. | PCP  1 (knowledge from guidelines)  12 (social influence of orthopaedic surgeon’s letter)  12 (social influence of NOF guidelines) |

**.**

Notes: use of italics highlights the section of text that justifies the code. A ‘T’ in front of the code indicates that the code is related solely to osteoporosis treatment with anti-resorptive therapy, and an ‘S’ solely to BMD scanning. The coding specified who the primary recipient of the intervention was, i.e. patient, PCP or pharmacist.

TDF Domains:

(1) Knowledge, (2) Skills, (3) Social/professional role and identity, (4) Beliefs about capabilities, (5) Optimism, (6) Beliefs about consequences, (7) Reinforcement, (8) Intentions, (9) Goals, (10) Memory, attention, and decision processes, (11) Environmental context and resources, (12) Social influences, (13) Emotion, (14) Behavioural regulation.
